# Effects of Spinach Extract and Licorice Extract on Growth Performance, Antioxidant Capacity, and Gut Microbiota in Weaned Piglets

**DOI:** 10.3390/ani14020321

**Published:** 2024-01-19

**Authors:** Jiahao Zhu, Jincong Lian, Haibin Deng, Junyi Luo, Ting Chen, Jiajie Sun, Yongliang Zhang, Yongan Yang, Pingxiang Liu, Qianyun Xi

**Affiliations:** 1 Guangdong Provincial Key Laboratory of Animal Nutrition Control, State Key Laboratory of Livestock and Poultry Breeding, National Engineering Research Center for Breeding Swine Industry, College of Animal Science, South China Agricultural University, No. 483 Wushan Road, Guangzhou 510642, China; zjh0926@stu.scau.edu.cn (J.Z.); lianjc@haid.com.cn (J.L.); denghaibin@stu.scau.edu.cn (H.D.); luojunyi@scau.edu.cn (J.L.); allinchen@scauedu.cn (T.C.); jiajiesun@scau.edu.cn (J.S.); zhangyl@scau.edu.cn (Y.Z.); 2 Elionnature Biotechnology Co., Ltd., No.16 Hengtong Road, Nanjing 210038, China; elionnature@163.com; 3 Guangdong Drive Bio-Tech Group Co., Ltd., No.9, Dengtang Industrial Zone, Guangzhou Road, Guangzhou 510642, China

**Keywords:** weaned piglet, spinach extract, licorice extract, growth performance, antioxidant capacity, gut microbiota

## Abstract

**Simple Summary:**

Anemia and oxidative stress are important factors affecting piglet growth performance. Spinach extract has been shown to improve anemia and licorice extract has also been shown to have antioxidant and anti-inflammatory properties. In this study, we investigated the effects of adding spinach extract and licorice extract to the diet on the production performance, serum biochemical indices, antioxidant capacity, and gut microbiota of weaned piglets. As a result, spinach extract improved the growth performance and hemoglobin level of weaned piglets, and licorice extract alleviated oxidative stress. In addition, we found that the combination of spinach extract and licorice extract was more effective. The combination of spinach extract and licorice extract improved the absorption of nutrients from the diets, and this effect may be, in part, related to the alteration of the gut microbiota.

**Abstract:**

Anemia and weaning stress are important factors affecting piglet growth performance. Spinach extract and licorice extract have been used to improve anemia and antioxidant capacity, respectively. However, whether they have synergistic effects has not been reported. To evaluate the effects of mixed spinach extract and licorice extract on growth performance, serum biochemistry, antioxidant capacity, and gut microbiota in weaned piglets, a total of 160 weaned piglets were randomly allotted to four treatments with four replications of 10 piglets each. The four treatments were as follows: control (CON) group (basal diet), spinach extract (SE) group (basal diet + 1.5 kg/t spinach extract), licorice extract (LE) group (basal diet + 400 g/t licorice extract), and spinach extract and licorice extract (MIX) group (basal diet + 1.5 kg/t spinach extract + 400 g/t licorice extract). The results showed that, compared with the CON group, diets supplemented with spinach extract and licorice extract significantly increased the average daily gain (*p* < 0.05), while considerably reducing the feed-to-gain ratio (*p* < 0.05). Moreover, the MIX group exhibited a significant up-regulation of serum total protein, globulin, albumin, glucose, and triglyceride levels in comparison to the CON group (*p* < 0.05). Meanwhile, both the anemia and antioxidant capacity of piglets were effectively improved. Notably, the MIX group achieved even better results than the individual supplementation in terms of enhancing growth performance, which could potentially be attributed to the increased abundance of the *Rikenellaceae_RC9_gut_group*. These results demonstrated that the supplementation of diets with spinach extract and licorice extract improves the absorption of nutrients from the diet and antioxidant capacity in weaned piglets.

## 1. Introduction

Anemia and oxidative stress in piglets have been the main problems affecting their growth performance. In the presence of anemia, the oxygen delivery capacity is reduced, which has a detrimental effect on the aerobic metabolism and utilization of glucose. Since energy is produced through oxygen, insufficient oxygen reduces the efficiency of ATP production, and the lactic acid produced by the anaerobic metabolism of glucose exacerbates the degree of anemia and the acid–base balance of the body [1]. Also, the ability to metabolize fatty acids aerobically is compromised [2]. In the meantime, oxidative stress has a significant negative impact on growth performance [3]. Oxidative stress leads to DNA, RNA, and protein damage and affects physiological processes such as tissue cell regeneration. Oxidative stress also causes inflammation in the small intestine and impairs nutrient absorption during the growing–finishing pig stage [4]; during the piglet stage, oxidative stress causes damage to the immune system, reduces liver and pancreatic islet function [5], and leads to the dysfunction of the immune, metabolic, and digestive systems in pigs [6].

There is a complex interaction between oxidative stress and anemia. Oxidative stress disrupts erythrocyte membranes, leading to a shortened lifespan in erythrocytes, which, in turn, leads to anemia [7]. The reduced or abnormal functioning of erythrocytes in the presence of iron deficiency also increases the risk of oxidative stress in the body [8]. Due to the insufficient number of red blood cells, oxygen delivery is reduced and cells do not receive enough oxygen and energy, which can lead to the formation of more free radical molecules in the body and cause more oxidative stress [9]. In addition, antioxidant capacity tends to be lower in anemic patients than in normal subjects, making them more vulnerable to oxidative stress. Antioxidant capacity refers to the ability to prevent the accumulation of free radicals and other harmful compounds, and the human body utilizes its own antioxidants to combat oxidative stress, which include vitamin C, vitamin E, and glutathione [10]. However, in patients with anemia, the levels of these antioxidants tend to be lower than normal, leading to more pronounced oxidative stress aggression. Decreased antioxidant capacity may be due to the fact that people with anemia eat an unbalanced diet and lack essential nutrients, resulting in the body’s inability to make enough antioxidants [11]. The co-addition of iron supplements and antioxidants to diets may be an effective means for improving both anemia and oxidative stress and, consequently, growth performance in weaned piglets.

Between the compounds that could be of potential interest to combine for avoiding these problems are spinach and licorice extracts. The main components of spinach extract are chlorophyll and vitamin B_12_, which promote hemoglobin synthesis as well as the development and maturation of red blood cells. It also participates in the body’s hematopoietic function, which, in turn, relieves anemia [12,13]. Licorice extract is rich in polyphenol compounds, a variety of flavonoids and other antioxidant components, which have powerful free radical scavenging abilities and antioxidant effects, and can prevent the animal intestinal tract from being damaged by the external environment and pathogenic microorganisms, safeguarding the animal’s health and growth and development [14,15]. Meanwhile, licorice acid, licorice flavonoids, and other active ingredients can regulate the inflammatory response in the animal body, thus reducing inflammatory damage [16]. Therefore, the objective of this study was to evaluate the effects of dietary supplementation with the mixture of spinach extract and licorice extract on the growth performance, blood biochemical indices, and gut microbiota of piglets, and to investigate whether the mixture of the two extracts is superior to adding them individually.

## 2. Materials and Methods

### 2.1. Materials

All Yorkshire × Landrace × Duroc weaned piglets were purchased from Huafeng pig farm (Chaozhou, Guangdong).

Spinach extract formulation (Quickhemo) was provided by Guangdong Drive Biotechnology Group Co., Ltd., (Guangzhou, China). The main components of spinach extract formulation (Quickhemo) were chlorophyll (40 g/kg) and vitamin B_12_ (40 mg/kg).

Licorice extract was provided by Elion Biotechnology Co., Ltd., (Nanjing, China). The main components of licorice extract formulation were polyphenol compounds and flavonoids.

### 2.2. Animals, Treatments, Housing, and Ethics Statement

A total of 160 healthy Yorkshire × Landrace × Duroc weaned piglets (28 days) were studied, with an initial body weight of 10.71 ± 1.07 kg and a ratio of males to females of 1:1. All piglets were randomly allotted to 4 treatments with 4 replications of 10 piglets in each pen. The 4 treatment groups were as follows: CON group: basal diet; SE group: basal diet + 1.5 kg/t spinach extract; LE group: basal diet + 400 g/t licorice extract; MIX group: basal diet + 1.5 kg/t spinach extract + 400 g/t licorice extract. The whole trial period lasted for 28 days, and the experiment ended when the piglets were 56 days old. Piglets were reared in the same barn with each replicate separated by a separate pen and allowed ad libitum access to feed and water. The temperature of the house was maintained at 25 °C on average. All animal experiments were accepted by the Animal Ethics Committee of South China Agricultural University, with permit number SYXK (Guangdong) 2022-0136. The principles for the care and use of animals authorized by the Animal Ethics Committee of South China Agricultural University were in accordance with all animal care procedures that were performed. Composition and nutrient levels of the basal diet are presented in Table 1.

### 2.3. Growth Performance and Diarrhea Rate

Pigs were fasted for 12 h the day before the start and end of the experimental period and then weighed to calculate the average daily gain (ADG). Feed consumption was recorded every 3 days. At the end of the experiment, average daily feed intake (ADFI) and the ratio of feed to gain (F/G) were calculated based on these values. Pig diarrhea incidents were observed and recorded every day, and the diarrhea rate was calculated by the number of diarrhea cases/(total number of pigs × trial days).

### 2.4. Sample Preparation and Analysis

At the beginning and end of the experimental period, we randomly select 2 piglets in each replicate. Blood (5 mL) was collected from the anterior vena cava, inserted into a coagulation tube, and stored at 4 °C. Hemoglobin concentration was measured immediately using hemoglobin measurement system. Serum was obtained by centrifugation at 1500× *g* for 30 min, and samples were stored at −80 °C.

Serum total protein (TP), albumin (ALB), globulin (GLB), urea nitrogen (BUN), glucose (GLU), triglyceride (TG), total cholesterol (TCH), alanine aminotransferase (ALT), glutamine aminotransferase (AST) serum superoxide dismutase (SOD), malondialdehyde (MDA), glutathione peroxidase (GSH-Px), and catalase (CAT) were determined by using a Microplate Reader, and the kits were purchased from Nanjing Jian Cheng Bioengineering Institute (Nanjing, China).

Feces from piglets were collected at the end of the experimental period; hands were washed and sterilized before collecting feces and sterile gloves were worn. One piglet per replicate was randomly selected to collect feces, which was caught by hand before it fell. A sampler was inserted into the deep center of the feces and gently twisted several times to take a small portion, which was loaded into a sterilized freezing tube. A total of 16 samples were obtained. Piglet feces were collected and stored at −80 °C for 16S rDNA sequencing.

### 2.5. Gut Microbiota Analysis

Total genome DNA was extracted from fecal content using the CTAB method. Bacterial 16S rRNA genes of distinct regions (V3–V4) were amplified using specific primers. PCR products were purified from a 2% agarose gel and isolated using a gel extraction kit. The sequencing was carried out on PacBio Sequel lle; QIIME was used for analysis. Samples were sent to Beijing Tsingke Biotech Co., Ltd., (Beijing, China).

### 2.6. Statistical Analysis

Data were analyzed by one-way ANOVA, followed by Dunn’s post hoc test using SPSS 22.0. In addition, nonparametric estimation was used to analyze microbial data. All the data are presented as mean ± SEM. *p* < 0.05 was considered to be statistically significant.

## 3. Results

### 3.1. Growth Performance

The effect of dietary spinach extract and licorice extract on the growth performance of piglets is presented in Table 2. Compared with the CON group, the ADG in the SE and MIX groups increased significantly (*p* < 0.05), while the F/G in the MIX group decreased significantly (*p* < 0.05). However, the growth performance of piglets in the LE group did not differ significantly from that of the control group (*p* > 0.05). There were no significant differences in the ADG and diarrhea rate between all treatment groups.

### 3.2. Serum Biochemistry

As shown in Table 3, the addition of spinach extract to the diet did not significantly affect serum biochemistry. We found that piglets in the LE group and MIX group had higher serum levels of GLB and GLU (*p* < 0.05) compared with the CON group. In addition, serum TP, ALB, and TG were increased in the MIX group compared with the CON group (*p* < 0.05). However, no significant differences were detected in serum levels of BUN and AST among the four groups of piglets (*p* > 0.05).

### 3.3. Antioxidant Properties

The serum antioxidant properties of piglets showed that there were no significant differences in the serum SOD and CAT among the four groups (*p* > 0.05, Table 4). Compared with the CON group, the serum GSH-Px levels in the other groups increased significantly (*p* < 0.05), while MDA decreased significantly in the SE and LE groups compared to the control group (*p* < 0.05). There was no significant difference in the MDA levels between the MIX and control groups (*p* > 0.05).

### 3.4. Hemoglobin Concentration

There was no significant difference in hemoglobin levels between the groups before the start of the experiment (*p* > 0.05, Table 5). However, at the end of the experiment, compared to the control group, the hemoglobin levels of the SE and MIX groups increased significantly (*p* < 0.05). There was no significant difference in the LE group compared to the control group (*p* > 0.05).

### 3.5. Gut Microbiota

**Figure 1:**
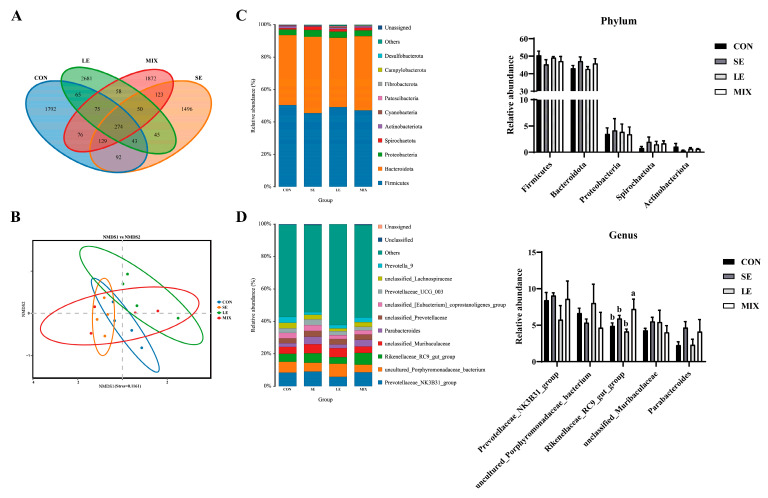
Regulation of gut microbiota by addition of spinach extract and licorice extract. (**A**) Venn diagrams and (**B**) NMDS analysis. The composition and relative abundance of the bacterial community in each sample at (**C**) the phylum and (**D**) the species levels (*n* = 4). Different superscripts within a row indicate significant differences (*p* < 0.05).

To investigate whether spinach extract and licorice extract play a regulatory role in the gut microbiota, we characterized the bacterial communities in our samples using 16S rRNA gene sequencing (Figure 1). Venn diagrams showed 8871 OTUs were identified, of which 274 OTUs were shared across all groups (Figure 1A). There was a multiple increase in microbial richness in the LE and MIX groups compared to the control group. In addition, the non-metric multidimensional scaling (NMDS) of the microbiota showed that the four treatment groups could be separated into distinct clusters (Figure 1B), although all groups did not differ in the relative abundance of microbial communities at the phylum (*p* > 0.05) (Figure 1C). Notably, at the species level, the relative abundance of *Rikenellaceae_RC9_gut_group* was significantly up-regulated in the MIX group compared to the control group (*p* < 0.05) (Figure 1D). However, no differential microbes were detected in the SE and LE groups compared to the control group.

## 4. Discussion

Symptoms of anemia are prevalent in the early growth stages of pigs, especially in the piglet stage, causing some economic losses in the pig industry. The current prophylactic treatments for anemia in piglets are ineffective, resulting in a high prevalence of anemic pigs after weaning. Iron and vitamin B_12_ are both hematopoietic raw materials, and deficiencies of iron and vitamin B_12_ are the main cause of anemia in weaned piglets. Several studies have shown that piglets that do not receive iron injections or iron supplements in their diets after birth deplete their body’s iron reserves, which leads to the development of iron deficiency anemia. This condition is characterized by reduced red blood cell counts, hemoglobin concentrations, and hematocrit, which leads to slow growth and development [17]. The addition of appropriate amounts of dietary iron supplements to the diet or iron supplementation by injection can be effective in improving piglet metabolism, growth performance, carcass parameters, and meat quality [18]. Vitamin B_12_ is one of the essential nutrients for the growth and development of piglets. If the feed is deficient in vitamin B_12_, it can also lead to iron deficiency anemia in piglets [19]. In this study, we selected spinach extract as a therapeutic agent to alleviate anemia in piglets, which is rich in vitamin B_12_. The results showed that the addition of spinach extract to the diet increased the daily weight gain of piglets and the mixture of spinach extract and licorice extract was more effective, provided that there was no difference in the ADFI of all groups. This may be due to the fact that spinach extract improved anemia in piglets and enhanced the intestinal absorption of nutrients from the diet. In addition, the co-addition of spinach and licorice extracts was also effective in reducing the feed-to-meat ratio. However, the addition of only licorice extract had no effect on the growth performance of piglets.

The serum biochemical indicator is a reflection of the body’s immune regulation and material metabolism, and is important for maintaining the homeostasis of the internal environment of the animal organism. Anemia and weaning stress both affect appetite, digestive, and absorptive function, which, in turn, affects nutrient utilization and causes pathological feeding problems [20]. Although the supplementation of spinach extract in the diet increased the hemoglobin of piglets, there was no significant effect on serum biochemical indices. Interestingly, serum TP, ALB, GLB, TG, TCH, and GLU were increased by the simultaneous addition of spinach extract and licorice extract. Serum TP and ALB concentrations reflect the status of protein metabolism, whereas serum GLB levels are closely related to immune status [21]. Serum TG and TCH levels are indicators of the body’s utilization of dietary lipids and fat metabolism, while serum GLU levels are used to reflect the body’s glucose metabolism. This suggests the increased absorption of dietary nutrients by piglets, which is consistent with the elevated ADG and reduced F/G.

Oxidative stress is a symptom caused by an imbalance between the antioxidant capacity of antioxidant molecules and the production of reactive oxygen species (ROS) in the animal body. Excess ROS in the body leads to the accumulation of pro-inflammatory substances, which, in turn, leads to further tissue damage [22]. The active components of licorice extract, including triterpenoids and flavonoids, have been shown to improve the anti-inflammatory, antioxidant, and immunomodulatory capacities of the animal organism [23]. The addition of 1 g/kg to 3 g/kg licorice extract to diets can significantly improve the growth performance and antioxidant indexes of chicks [24]. The application of licorice in pig feed can effectively improve the immune function of pigs and can replace the use of antibiotics to a certain extent [25]. Indeed, in the current study, GSH-Px activity was significantly improved by adding spinach extract or licorice extract. At the same time, the activity of MDA was decreased.

Hemoglobin concentration has an important effect on both the growth performance and survival of pigs. It has been found that hemoglobin concentration in sows affects piglet survival, and the level of hemoglobin can be used as one of the indicators for the genetic selection of pigs [26]. However, it is hard to increase hemoglobin concentration, and some studies have also found that the addition of various organic iron mixtures to the diet of sows did not significantly increase hemoglobin concentration [27]. Adding dietary iron supplements to anemic piglet diets can effectively improve the hematological indicators and increase the hemoglobin levels of piglets, thereby improving growth performance and health [28]. In our experiment, piglets were initially anemic (hemoglobin concentration < 90 g/L) before starting the trial, but their hemoglobin concentrations significantly increased at the end of the trial in both the SE group and the MIX group compared to the control group, indicating that the appropriate addition of spinach extract can increase the hemoglobin concentration of piglets and contribute to their growth and health.

Gut microbes also play an important role in maintaining intestinal health in weaned piglets. The microbiota effectively prevents the invasion of exogenous pathogens and harmful substances, and also promotes the absorption and utilization of nutrients by the organism [29]. Stress in weaned piglets can also act on the gut, resulting in disturbances in the intestinal microbiota and a decrease in growth performance [30]. The addition of spinach extract and licorice extract may also indirectly modulate growth performance and the immune system of pigs by affecting the gut microbiota. Spinach is rich in vitamin B_12_, which can effectively improve the structure of intestinal flora and short-chain fatty acid levels, and the relative abundance of *Firmicutes* also increased [31]. Similarly, chlorophyll performs the same function [32]. The addition of licorice polysaccharides to broiler diets promotes the secretory activity of cuprocytes, alters the gut microbial composition, and increases species diversity and abundance [33]. Feeding Zi geese with a compound herbal remedy containing licorice significantly increased the proportion of beneficial bacteria in abundance in their cecum [34]. Similar to these results, we found that the co-addition of spinach extract and licorice extract increased the relative abundance of *Rikenellaceae_RC9_gut_group* at the genus level. It has been found that the *Rikenellaceae_RC9_gut_group* is able to ferment polysaccharides that are unabsorbed in the host intestine to produce short-chain fatty acids (SCFAs) [35], which corresponds to the improved growth performance of piglets in the co-addition of spinach extract and licorice group.

## 5. Conclusions

In conclusion, both spinach extract alone and the mixture of spinach extract and licorice extract improved the growth performance of piglets, but the mixture of spinach extract and licorice extract was better than the spinach extract alone. The results of the serum biochemical indices showed that mixing spinach extract with licorice extract could improve the absorption of nutrients in the diet, which might be related to the increase in beneficial bacteria, *Rikenellaceae_RC9_gut_group*. In terms of serum antioxidant indices, both spinach extract and licorice extract improved the serum antioxidant capacity of piglets.

## Figures and Tables

**Table 1:** Composition and nutrient levels of the basal diet for piglets (air-dry basis, %).

Ingredients	Content %	Nutrient Levels ^2^	Content
Corn	60.1	Crude protein	19.86
Soybean meal	19.6	Ether extract	4.03
Extruded soybean	8.0	Calcium	0.86
Fish meal	3.0	Total phosphorus	0.67
Whey powder	3.0	Lysine	1.43
Soy oil	2.0	Methionine + Cystine	0.78
NaCl	0.3	Threonine	0.86
Premix ^1^	4.0	Tryptophan	0.24
Total	100	Digestible energy, MJ/kg	12.98

^1^ The premix provided the following per kg of diets: VA, 11,000 IU; VD_3_, 1000 IU; VE, 15 IU; VK, 10 mg; VB_1_, 0.6 mg; VB_2_ 0.6 mg; VB_6_ 1.5 mg; VB_12_ 0.03 mg; VB_4_ 800 mg; biotin, 6 mg; nicotinic acid, 10 mg; folic acid, 0.8 mg; Fe (as ferrous sulfate), 110 mg; Zn (as zinc sulfate), 100 mg; Cu (as copper sulfate), 20 mg; Mn (as manganese sulfate), 40 mg; Co (as cobalt chloride), 0.3 mg; I (as potassium iodide), 0.4 mg; Se (as sodium selenite), 0.3 mg. ^2^ Digestible energy was a calculated value, while the others were measured values.

**Table 2:** Effects of dietary spinach extract or licorice extract supplementation on growth performance and diarrhea rate in weaned piglets (*n* = 4).

Item ^1^	Treatment ^2^				*p*-Value
	CON	SE	LE	MIX	
Initial weight, kg	11.31 ± 0.41	10.3 ± 0.58	10.74 ± 0.73	10.47 ± 0.45	0.610
Final weight, kg	20.74 ± 0.55 ^ab^	21.65 ± 0.99 ^ab^	19.94 ± 0.29 ^b^	22.67 ± 0.48 ^a^	0.040
ADG, kg/d	0.34 ± 0.02 ^b^	0.41 ± 0.02 ^a^	0.33 ± 0.02 ^b^	0.44 ± 0.01 ^a^	0.006
ADFI, kg/d	0.67 ± 0.02	0.70 ± 0.03	0.68 ± 0.01	0.71 ± 0.04	0.684
F/G	2.03 ± 0.16 ^ab^	1.74 ± 0.08 ^bc^	2.11 ± 0.11 ^a^	1.64 ± 0.09 ^c^	0.020
Diarrhea rate, %	1.48 ± 0.21	1.33 ± 0.23	1.43 ± 0.25	1.28 ± 0.19	0.913

^1^ ADG = average daily gain; ADFI = average daily feed intake; F/G = ratio of feed to gain. ^2^ CON group: basal diet; SE group: basal diet + 1.5 kg/t spinach extract; LE group: basal diet + 400 g/t licorice extract; MIX group: basal diet + 1.5 kg/t spinach extract + 400 g/t licorice extract. ^a,b,c^ Different superscripts within a row indicate significant differences (*p* < 0.05).

**Table 3:** Effects of dietary spinach extract or licorice extract supplementation on serum biochemistry levels in weaned piglets (*n* = 4).

Item ^1^	Treatment ^2^				*p*-Value
	CON	SE	LE	MIX	
TP (g/L)	53.18 ± 2.87 ^b^	57.14 ± 1.99 ^b^	57.60 ± 0.22 ^b^	66.57 ± 1.93 ^a^	0.009
GLB (g/L)	17.27 ± 1.09 ^b^	18.75 ± 0.83 ^b^	21.98 ± 1.19 ^a^	21.88 ± 0.87 ^a^	0.021
ALB (g/L)	35.91 ± 3.86 ^b^	38.39 ± 1.25 ^ab^	35.62 ± 1.89 ^b^	44.69 ± 1.27 ^a^	0.051
BUN (mmol/L)	3.22 ± 0.22	3.09 ± 0.05	3.47 ± 0.22	3.33 ± 0.25	0.689
GLU (mmol/L)	4.58 ± 0.45 ^c^	6.01 ± 0.64 ^bc^	6.78 ± 0.66 ^ab^	8.43 ± 0.37 ^a^	0.005
TG (mmol/L)	0.73 ± 0.08 ^b^	0.52 ± 0.06 ^b^	0.52 ± 0.07 ^b^	1.00 ± 0.05 ^a^	0.002
TCH (mmol/L)	1.74 ± 0.17 ^ab^	1.65 ± 0.09 ^ab^	1.55 ± 0.11 ^b^	2.06 ± 0.12 ^a^	0.089
ALT (U/L)	3.99 ± 0.25 ^ab^	2.80 ± 0.22 ^b^	3.02 ± 0.49 ^b^	5.06 ± 0.49 ^a^	0.011
AST (U/L)	4.28 ± 0.56	4.59 ± 0.58	6.71 ± 1.61	6.88 ± 0.60	0.117

^1^ TP = total protein; ALB = albumin; GLB = globulin; BUN = urea nitrogen; GLU = glucose; TG = triglyceride; TCH = total cholesterol; ALT = alanine aminotransferase; AST = aspartate aminotransferase. ^2^ CON group: basal diet; SE group: basal diet + 1.5 kg/t spinach extract; LE group: basal diet + 400 g/t licorice extract; MIX group: basal diet + 1.5 kg/t spinach extract + 400 g/t licorice extract. ^a,b,c^ Different superscripts within a row indicate significant differences (*p* < 0.05).

**Table 4:** Effects of dietary spinach extract or licorice extract supplementation on serum antioxidant function in weaned piglets (*n* = 4).

Item ^1^	Treatment ^2^				*p*-Value
	CON	SE	LE	MIX	
SOD (U/mL)	211.66 ± 10.88	202.01 ± 11.86	222.51 ± 11.74	211.52 ± 8.58	0.650
MDA (nmol/mL)	4.11 ± 0.11 ^a^	2.84 ± 0.53 ^b^	2.35 ± 0.45 ^b^	3.48 ± 0.12 ^ab^	0.004
GSH-Px (U/mL)	952.18 ± 21.19 ^b^	1396.39 ± 34.62 ^a^	1248.12 ± 66.49 ^a^	1384.36 ± 78.57 ^a^	0.002
CAT (U/mL)	5.13 ± 0.19	4.85 ± 0.93	5.21 ± 0.34	4.53 ± 0.62	0.901

^1^ SOD = superoxide dismutase; MDA = malondialdehyde; GSH-pX = glutathione peroxidase; CAT = catalase. ^2^ CON group: basal diet; SE group: basal diet + 1.5 kg/t spinach extract; LE group: basal diet + 400 g/t licorice extract; MIX group: basal diet + 1.5 kg/t spinach extract + 400 g/t licorice extract. ^a,b^ Different superscripts within a row indicate significant differences (*p* < 0.05).

**Table 5:** Effects of dietary spinach extract or licorice extract supplementation on hemoglobin concentration in weaned piglets (*n* = 5).

Item	Treatment ^1^				*p*-Value
	CON	SE	LE	MIX	
Initial concentration (g/L)	83.40 ± 4.82	82.20 ± 5.07	85.00 ± 3.48	84.20 ± 2.92	0.969
Final concentration (g/L)	79.00 ± 1.48 ^b^	87.60 ± 1.21 ^a^	82.80 ± 2.20 ^b^	88.40 ± 1.03 ^a^	0.002

^1^ CON group: basal diet; SE group: basal diet + 1.5 kg/t spinach extract; LE group: basal diet + 400 g/t licorice extract; MIX group: basal diet + 1.5 kg/t spinach extract + 400 g/t licorice extract. Severe anemia (hemoglobin concentration < 90 g/L), anemic (hemoglobin concentration < 110 g/L). ^a,b^ Different superscripts within a row indicate significant differences (*p* < 0.05).

## Data Availability

The 16S rDNA gene sequence data have been deposited in the NCBI BioProject database (https://www.ncbi.nlm.nih.gov/bioproject/, accessed on 5 December 2023) under accession numbers PRJNA1048749.
