# Correction: Zhu et al. Effects of Spinach Extract and Licorice Extract on Growth Performance, Antioxidant Capacity, and Gut Microbiota in Weaned Piglets. *Animals* 2024, *14*, 321

**DOI:** 10.3390/ani16020271

**Published:** 2026-01-16

**Authors:** Jiahao Zhu, Jincong Lian, Haibin Deng, Junyi Luo, Ting Chen, Jiajie Sun, Yongliang Zhang, Yongan Yang, Pingxiang Liu, Qianyun Xi

**Affiliations:** 1 Guangdong Provincial Key Laboratory of Animal Nutrition Control, State Key Laboratory of Livestock and Poultry Breeding, National Engineering Research Center for Breeding Swine Industry, College of Animal Science, South China Agricultural University, No. 483 Wushan Road, Guangzhou 510642, China; zjh0926@stu.scau.edu.cn (J.Z.); lianjc@haid.com.cn (J.L.); denghaibin@stu.scau.edu.cn (H.D.); luojunyi@scau.edu.cn (J.L.); allinchen@scauedu.cn (T.C.); jiajiesun@scau.edu.cn (J.S.); zhangyl@scau.edu.cn (Y.Z.); 2 Elionnature Biotechnology Co., Ltd., No.16 Hengtong Road, Nanjing 210038, China; elionnature@163.com; 3 Guangdong Drive Bio-Tech Group Co., Ltd., No.9, Dengtang Industrial Zone, Guangzhou Road, Guangzhou 510642, China

In the original publication [1], there was a mistake in Table 2 as published. The correct caption for row 5 is “ADG” and the correct caption for row 6 is “ADFI”. “ADG” is also incorrectly described as “ADFI” in the presentation of Section 3.1 and in the last line of the second paragraph of the Discussion section. “ADFI” is also incorrectly described as “ADG” in the first paragraph of the Discussion section. There is also an error in the data for the CON group in the third row of Table 2, where the correct data is “0.34”. The corrected Table 2 appears below. The authors state that the scientific conclusions are unaffected. This correction was approved by the Academic Editor. The original publication has also been updated.

## Figures and Tables

**Table 2:** Effects of dietary spinach extract or licorice extract supplementation on growth performance and diarrhea rate in weaned piglets (*n* = 4).

Item ^1^	Treatment ^2^				*p*-Value
	CON	SE	LE	MIX	
Initial weight, kg	11.31 ± 0.41	10.3 ± 0.58	10.74 ± 0.73	10.47 ± 0.45	0.610
Final weight, kg	20.74 ± 0.55 ^ab^	21.65 ± 0.99 ^ab^	19.94 ± 0.29 ^b^	22.67 ± 0.48 ^a^	0.040
ADG, kg/d	0.34 ± 0.02 ^b^	0.41 ± 0.02 ^a^	0.33 ± 0.02 ^b^	0.44 ± 0.01 ^a^	0.006
ADFI, kg/d	0.67 ± 0.02	0.70 ± 0.03	0.68 ± 0.01	0.71 ± 0.04	0.684
F/G	2.03 ± 0.16 ^ab^	1.74 ± 0.08 ^bc^	2.11 ± 0.11 ^a^	1.64 ± 0.09 ^c^	0.020
Diarrhea rate, %	1.48 ± 0.21	1.33 ± 0.23	1.43 ± 0.25	1.28 ± 0.19	0.913

^1^ ADG = average daily gain; ADFI = average daily feed intake; F/G = ratio of feed to gain. ^2^ CON group: basal diet; SE group: basal diet + 1.5 kg/t spinach extract; LE group: basal diet + 400 g/t licorice extract; MIX group: basal diet + 1.5 kg/t spinach extract + 400 g/t licorice extract. ^a,b,c^ Different superscripts within a row indicate significant differences (*p* < 0.05).
